# Molecular Proteomics and Signalling of Human Platelets in Health and Disease

**DOI:** 10.3390/ijms22189860

**Published:** 2021-09-13

**Authors:** Jingnan Huang, Pengyu Zhang, Fiorella A. Solari, Albert Sickmann, Angel Garcia, Kerstin Jurk, Johan W. M. Heemskerk

**Affiliations:** 1 Leibniz Institut für Analytische Wissenschaften—ISAS-e.V., 44139 Dortmund, Germany; pengyu.zhang@isas.de (P.Z.); fiorella.solari@isas.de (F.A.S.); sickmann@isas.de (A.S.); 2 Department of Biochemistry, CARIM, Maastricht University, 6229 ER Maastricht, The Netherlands; 3 Center for Research in Molecular Medicine and Chronic Diseases (CIMUS), Universidade de Santiago de Compostela, 15706 Santiago de Compostela, Spain; angel.garcia@usc.es; 4 Center for Thrombosis and Haemostasis, University Medical Center of the Johannes Gutenberg University Mainz, 55131 Mainz, Germany; kerstin.jurk@unimedizin-mainz.de; 5 Medizinische Fakultät, Medizinische Proteom-Center, Ruhr-Universität Bochum, 44801 Bochum, Germany; 6 Department of Chemistry, College of Physical Sciences, University of Aberdeen, Aberdeen AB24 3FX, UK

**Keywords:** platelets, post-translational modification, proteins, signalling, receptors

## Abstract

Platelets are small anucleate blood cells that play vital roles in haemostasis and thrombosis, besides other physiological and pathophysiological processes. These roles are tightly regulated by a complex network of signalling pathways. Mass spectrometry-based proteomic techniques are contributing not only to the identification and quantification of new platelet proteins, but also reveal post-translational modifications of these molecules, such as acetylation, glycosylation and phosphorylation. Moreover, target proteomic analysis of platelets can provide molecular biomarkers for genetic aberrations with established or non-established links to platelet dysfunctions. In this report, we review 67 reports regarding platelet proteomic analysis and signalling on a molecular base. Collectively, these provide detailed insight into the: (i) technical developments and limitations of the assessment of platelet (sub)proteomes; (ii) molecular protein changes upon ageing of platelets; (iii) complexity of platelet signalling pathways and functions in response to collagen, rhodocytin, thrombin, thromboxane A_2_ and ADP; (iv) proteomic effects of endothelial-derived mediators such as prostacyclin and the anti-platelet drug aspirin; and (v) molecular protein changes in platelets from patients with congenital disorders or cardiovascular disease. However, sample sizes are still low and the roles of differentially expressed proteins are often unknown. Based on the practical and technical possibilities and limitations, we provide a perspective for further improvements of the platelet proteomic field.

## 1. Introduction

Platelets circulate as anucleated cells in the blood, where they are kept in a resting state by the vascular endothelium producing platelet inhibitors [1,2]. Alongside other functions, not addressed in this paper, platelets are of fundamental importance in primary and secondary haemostasis and arterial thrombosis [3]. An extensive network of molecular signal transduction processes in platelets allows their fast adhesion and secretion upon injury of the vessel wall or damage of an atherosclerotic plaque, and hence allows the formation of a rapidly growing thrombus [4,5].

Because of their anucleate structure, gene transcription and ribosomal translation activities are restricted in platelets [6,7], resulting in a relatively stable proteome [8]. Given this, mass-spectrometry-based proteomic analyses can be a valuable tool to assess the molecular build-up especially of these out-differentiated cells.

## 2. Overview of Platelet Proteomic Literature

In the present paper, we provide a topical overview of how technical developments in the mass-spectrometric technologies are contributing to our knowledge of the basic proteome of freshly isolated and stored platelets, as well as of platelets stimulated via key receptor-dependent signalling mechanisms. Details of the 67 published platelet proteomic analyses are provided in Table S1 (search terms PubMed 2021: platelet proteomics, excluding non-human, not original protein lists and reviews). Extracted key characteristics per section are indicated in Table 1.

**Figure 1:**
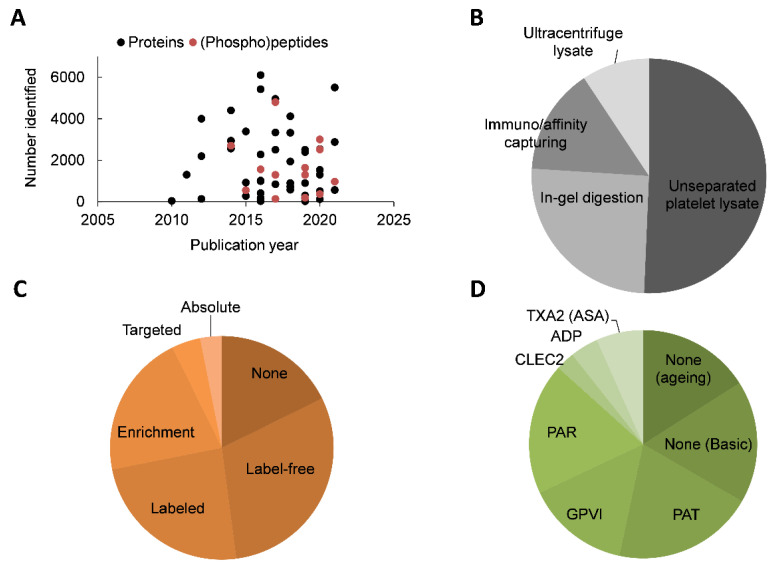
Overview of 67 platelet proteomic publications. (**A**), Numbers of identified proteins and (phospho)peptides in human platelets in publications over the years. (**B**), Distribution profile of proteomic papers for types of platelet preparations. Half of the collected papers (49%) used trypsin treatment of unseparated platelet lysates; others used prior separation by gel electrophoresis and in-gel digestion (26%), immuno/affinity capturing (15%) or ultracentrifugation enrichment (9%). (**C**), Distribution profile of proteomic quantification type. In 67 publications, 20% used no other method, 23% used stable isotope labelling, 30% used label-free quantification, 22% employed special peptides enrichments and 5% employed targeted (4%) or absolute quantification (1%) methods. (**D**), Distribution profile of type of (stimulated) platelets used in 67 publications. Most papers used healthy donor platelets without agonist (33%), patient platelets (PAT, 20%) or platelets stimulated via GPVI (15%) or PARs (17%). Rarer was analysis of platelets stimulated via CLEC-2 (3%), ADP (4%) or TXA_2_/aspirin (7%).

With an estimated size of ~10 k unique proteins in nucleated cells [9,10], proteome studies of human platelets have identified variable numbers of 2–6 k proteins (Figure 1A). From the 67 registered studies, including six papers with two categories (Table S1), many (48%) used isolated, washed platelets of high purity (99–99.99%). Given the small volume of platelets (9–11 fL) [2] and an abundant open canicular system, this purity cannot exclude contamination with proteins from plasma, red blood cells and leukocytes, which may affect study outcomes [11]. A minority of the studies investigated platelet releasates, ultracentrifuged platelet extracellular vesicles, or immuno-affinity fractions from platelets, with the latter providing lower numbers of proteins [12]. The early procedure of two-dimensional gel separation of lysed platelets is still in use in some laboratories (12 papers), although this method is now mostly replaced by bottom-up LC-MS/MS analysis without gel separation.

As the most common protein digestion method, about half of the collected papers (49%) use trypsin treatment of total platelet lysates (Figure 1B). The usual sample workup is trichloroacetic acid and/or acetone precipitation and filter-aided sample preparation. Many of the 67 publications describe one or more additional sample treatment steps before uploading onto a column. These include stable isotope labelling (isobaric tags for relative and absolute quantification, iTRAQ or TMT 23%), label-free quantification (30%), enrichment (22%, for phosphopeptides, N-terminal or glycopeptides) or targeted or absolute quantification (5%) (Figure 1C). In recent years, label-free quantification has become possible with state-of-the-art mass spectrometers, but this method is demanding for subsequent data analysis [13,14]. As we detail below (Section 11), novel technical advances are gradually appearing in papers, such as label-free quantification methods, data-independent acquisition, targeted analysis with biomarker peptide references and well-plate-based sample workup.

The majority of the 67 publications state some study limitations. The most mentioned are: low peptide coverage linked to low protein abundance, complex spectral data analysis and missing (hydrophobic) peptide sequences [15]. It has been recognised that inter-lab differences in fractionation and instrumentation limit the comparison of the platelet (phospho)proteomes, published by various groups [16,17]. So far, all studies have examined low sample sizes (platelets from few subjects of mostly unknown gender); hence, questions about inter-subject variation and comparisons of subject cohorts remain to be answered [18,19,20].

**Figure 2:**
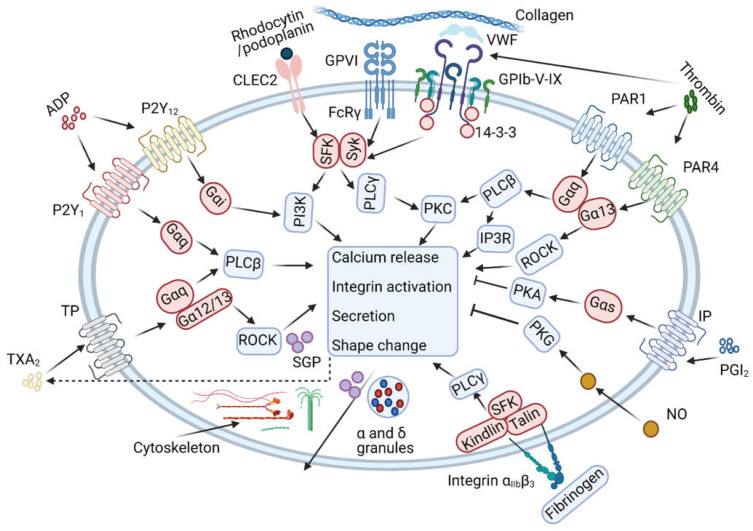
General overview of platelet signalling pathways. Overview of key platelet signalling and responses via key platelet receptors and agonists, examined by platelet proteomic analysis. Indicated are signalling via the collagen receptor glycoprotein (GP)VI, VWF receptor GPIb-V-IX; the proteinase-activated receptor (PAR)1 and PAR4 for thrombin; the podoplanin and rhodocytin receptor C-type lectin receptor 2 (CLEC2); the ADP receptors P2Y_1_ and P2Y_12_; thromboxane (TX)A_2_ mimetic U46619 (pathway inhibited by aspirin); integrin α_IIb_β_3_ outside/in signalling; actin and tubulin cytoskeletons; platelet-inhibiting prostacyclin I_2_ (PGI_2_) and nitric oxide (NO); and α-granule and δ-granule secretion. Other abbreviations: Gα, heterotrimeric G proteins; PKA, PKC, PKG, protein kinases A, C, G; PLC, phospholipase C; SGP, small GTP-binding proteins; SFK, Src-family kinase. Refs. [2,5,21]. Originally created with BioRender.com accessed on 10 July 2021.

Since 2011, with the improvement of mass spectrometric approaches, gradual progress has been made in unravelling the ′basic’ platelet proteome and post-translational modifications (Figure 1D). Markedly, there are a substantial number of studies interested in the ageing-related proteomic changes of in vitro stored platelets. The remaining works studied aspects of platelet signalling in response to key platelet receptors and agonists. As schematised in Figure 2, these concern signalling pathways via the collagen receptor glycoprotein VI (GPVI, ligands: collagen, collagen-related peptide CRP and convulxin); furthermore, signalling via the thrombin (co)receptors GPIbα, PAR1 and PAR4 (ligands: thrombin, thrombin-receptor-activating peptides); the C-type lectin receptor CLEC-2 (ligands: podoplanin, rhodocytin); the ADP receptors P2Y_12_ and P2Y_1_; the thromboxane A_2_ (TXA_2_) mimetic U46619 (a pathway inhibited by aspirin); and the platelet-inhibiting agents prostacyclin and nitric oxide.

## 3. Basic Platelet Proteome

Based on genome-wide platelet transcriptome information, the theoretical platelet proteome (i.e., the number of expressed protein-encoding genes) is now estimated at 14.8 k proteins. As far as we understand it now, particularly abundant in the identified platelet proteome are mitochondrial, metabolic, signalling/adaptor and transcription proteins [11]. In the past decade, significant progress has been made in establishing the protein composition of platelets freshly isolated from human blood samples. Numbers of proteins identified by mass spectrometry and by label-free analysis have increased from 1.3 k in 2011 [22] to 5.4 k [23], of which 3.7 k are with estimated copy numbers [24]. Analysis of the 500 proteins with the highest copy numbers indicated the highest abundance of proteins in the actin and microtubule cytoskeletons, the α-granules, involved in signalling, and (regulating) small GTPases [25]. Specific analyses indicated the abundant presence of nearly complete 20 S and 26 S proteasomes, implicating regular protein degradation [26]. A study of small and large platelets derived from the same healthy donor revealed that 80 proteins (9%) differed in abundance [27]. These included signalling proteins, but also several plasma proteins (Table 2).

Several mass spectrometry studies have examined the post-translational modifications of platelet proteins. For the identification of phosphoproteins, commonly a TiO_2_ sample enrichment step was used, often in combination with a calibration label, probe or antibody [8,20,25]. Reported changes in protein phosphorylation are discussed in the paragraphs below. In another enrichment protocol of platelet membranes, 0.2 k palmitoylated proteins could be identified, among which was the TREM-like transcript-1 (TLT1) surface receptor [22]. Distinct enrichment protocols of specific platelet peptides identified 0.2 k lysine methylations [32] and 1.6 k neo N-termini [28,33], indicating that protein methylation and cleavage play vital roles in normal platelet function. In a study of unclear physiological significance, a 5.4 k platelet proteome was reported, of which ~10% was regulated by the serotonin antagonist sarpogrelate [23]. However, the precise role of serotonin in platelet activation still needs to be defined [34]. Reported limitations of the various studies are the unclear relations of the newly identified platelet proteins and the low numbers of proteomes analysed (Table 1).

## 4. Proteome Changes in Ageing Platelets

The preferred way to store (ageing) platelets for best preservation of their functions after transfusion is a long-standing issue [35]. Transcriptome-based studies indicated that freshly isolated platelets have a limited capacity for protein synthesis, along with ongoing degradation of RNA species [36]. Commonly, platelet concentrates for transfusion purposes can be stored for multiple days, after which the platelets start to lose functional properties, a phenomenon known as platelet storage lesion [37]. To investigate the cause of this lesion, multiple studies have been carried out on the protein changes of ageing platelets. Since 2018, label-free quantification methods have been used in particular (Table S1).

An early paper assessed the extent of protein degradation by determining N-terminal methionines (i.e., formed by acetylation or endo-proteolysis) [33]. It was reported that the majority (77%) of 2.9 k identified proteins contained neo N-termini, which suggested extensive proteolytic processing during platelet storage. Matrix metalloproteinases were found to play an important role in this neo N-terminus formation, pointing to a continued protein cleavage in the platelet granules and in other membrane vesicles. In a platelet apheresis intervention protocol to enrich younger circulating platelets (one donor and 1.0 k identified proteins, hence low power), it was reported that endocytosis- and cytoskeleton-related proteins changed with the platelet age [38].

Some studies compared the ageing of platelets in different storage media, using 2D gel-based or pre-labelled proteomic techniques [39]. While differences in several proteins were observed between storage arms of the studies, the relationship with changes in platelet functions were not always clear. Two quantitative proteomic analyses indicated that, with increasing storage time, modified proteins especially had a role in degranulation [13,40]. Platelets stored in the cold at 2–6 °C were found to express reduced levels of glycoproteins and increased levels of surface activation markers, likely due to stimulation of glycoprotein shedding [41], but the cold storage did not affect platelet viability [40]. An induction of platelet degranulation was caused by pathogen inactivation technologies (e.g., with Mirasol) before storage [42].

A number of groups have been searching for platelet proteins that can explain adverse transfusion reactions affecting the patient’s health after the transfusion of ageing platelets. Regarding pathogen inactivation, treatment of platelet concentrates with riboflavin and ultraviolet light for two days—producing reactive oxygen species—resulted in slight increases in oxidised peptides, when compared to the 18% of 9.4 k identified platelet peptides that were oxidised anyway [43,44]. Proteomic studies on extracellular vesicles formed from ageing platelets revealed that metabolic proteins (e.g., glycolysis and lactate production) and proinflammatory cytokines (CCL5, PF4) were upregulated upon ageing [45]. A comparison of pooled platelet concentrates and single-donor apheresis platelets—both of which are preparations that occasionally trigger adverse transfusion reactions—revealed a partly common set of proteome changes (i.e., granular and mitochondrial proteins). On the other hand, signalling pathway analysis also revealed some differences: altered integrin α_IIb_β_3_ signalling in the pooled concentrates and acute phase response pathways in the apheresis platelets [46,47].

## 5. Collagen Receptor Glycoprotein VI (GPVI)

GPVI is a key collagen and fibrin receptor on platelets (3–4 k copies per cell), which signals via a cascade of protein tyrosine kinases, leading to strong platelet activation [48,49]. GPVI interaction with subendothelial collagens induces arterial thrombus formation upon vascular injury, as confirmed in multiple in vivo studies with genetically modified mice and in microfluidics studies with mouse or human blood [50,51]. At high wall shear rates, this interaction is preceded by platelet adhesion to von Willebrand factor (VWF), which avidly binds to collagens, via the GPIb-V-IX complex [52].

Platelet GPVI binding to collagens and to a lesser extent fibrin induces a profound activation cascade, initiated by tyrosine phosphorylation of immunoreceptor tyrosine-based activation motifs (ITAM) in its co-receptor Fc receptor γ-chain (FcRγ), which ultimately leads to platelet aggregation, secretion and procoagulant activity [5,53]. The signalling pathway involves Src-family kinases (SFK) and the spleen tyrosine kinase Syk, which triggers the activation of multiple signalling enzymes and adaptors, such as Btk/Tec family kinases, LAT, SLP76, phospholipase C γ2 (PLCγ2) and PI3 K isoforms (Figure 2) [54]. As GPVI-deficient patients experience variable but mostly not severe bleeding episodes, this underscores the concept of a more prominent role of GPVI in arterial thrombosis than in haemostasis [55,56].

First studies using gel spots to identify phosphorylated signalling targets resulted in only small sets of differentially regulated proteins linked to GPVI-induced platelet activation (Table S1) [57,58]. However, in a recent GPVI phosphoproteome paper, >3.0 k phosphorylation events (>1.3 k proteins) were identified by tandem mass tag (TMT) labelling and triple-stage tandem mass spectrometry. With literature-guided causal inference tools, more than 0.3 k site-specific signalling proteins could be obtained, among which were key and emerging GPVI effectors (i.e., FcγR, Syk, PLCγ2 and DAPP1) [59]. Interesting is the phosphorylation identification, downstream of GPVI, of a wide panel of small GTP-binding proteins (Ras and Rab GTPases) and activities of MAPK pathways [59]. The higher GPVI-induced platelet aggregation in patients with myocardial infarction was accompanied by increased phosphorylation of several of these signalling proteins [60]. Similarly, ~0.2 k basal phosphorylation sites were upregulated in platelets from obese patients, which were associated with augmented platelet adhesion to collagen [61].

Proteomic studies also focussed on other post-translation modifications. Thus, a procedure to assess reversible protein acetylation found 0.6 k acetyl-lysine residues (0.3 k proteins) serving as substrates for lysine acetyltransferases, which seem to be regulated in response to GPVI activation and subsequent cytoskeletal changes [62]. Another study detected 0.8 k extra-cytosolic N-linked glycosylation sites with some of these regulated by GPVI [63]. This agrees with the finding that glycan glycosylation plays a role in platelet–collagen interaction [64]. In GPVI-stimulated platelets, it appeared that 0.9 k out of 1.6 k ubiquitinylated peptides (corresponding to 0.7 k proteins, including Syk, filamin and integrins) were upregulated, implicating a profound role of GPVI in the ubiquitin protein degradation pathway [65]. To what extent proteins are degraded after GPVI stimulation is still unclear.

Lipidomic mass spectrometry of complexed phosphoinositides and bound proteins showed that upon PI3K activation, many proteins can interact with 3-phosphorylated phosphoinositides through their PH domains, among which is the new adaptor protein DAPP1 [66]. Membrane lipid rafts of GPVI-stimulated platelets were also analysed in detail [67]. Oligophrenin as a small GTPase-binding protein regulator was furthermore recognised as a functional target of the GPVI-induced tyrosine kinase cascade [68]. To what extent secondary mediators (ADP, TXA_2_) contribute to the GPVI-induced proteome changes has not systematically been investigated.

Stimulation via GPVI—enforced by thrombin—is an exemplary condition to evoke platelet procoagulant activity [69,70]. This response induced by a prolonged cytosolic Ca^2+^ elevation, ballooning and phosphatidylserine exposure allows the assembly and activation of coagulation factors on the platelet surface [71]. Proteomic studies have contributed to better understand these GPVI-mediated platelet responses. This held for platelets from a patient with Scott syndrome, i.e., a rare bleeding disorder with autosomal recessive mode of transmission. In the patient’s platelets, lacking phosphatidylserine externalisation due to a mutation in the ANO6 gene, the corresponding protein was found to be absent along with a reduction in calpain-like proteins [28]. The latter observation could explain a reduced cytoskeleton cleavage and a prolonged agonist-induced phosphorylation pattern (1.6 k phosphopeptides) in response to GPVI + PAR stimulation or ionomycin. Of the identified proteins, 6% were found to be up- or downregulated in the patient platelets, among which were several membrane glycoproteins (Table 2).

## 6. Signalling C-Type Lectin Receptor 2 (CLEC-2)

The hem-ITAM-linked receptor CLEC-2 triggers multiple platelet activation processes, with podoplanin as a known ligand in the lymphatic system, but no clear ligand in the blood or healthy blood vessels. On the other hand, platelet CLEC-2 has been recognised as a key receptor in thrombo-inflammatory disorders [72]. Similarly to GPVI, activation of CLEC-2 triggers a tyrosine kinase cascade, resulting in SFK and Syk activation via a hem-ITAM domain (Figure 2). Downstream signalling routes involve PLCγ2, Tec and PI3K family members [73,74]. A laboratory ligand of CLEC-2 is the snake venom rhodocytin.

Several platelet proteomic studies have helped to further resolve CLEC-2-induced signalling pathways. In rhodocytin-stimulated platelets, following gel separation and phosphoprotein enrichment, 0.1 k proteins of the broad signalosome were identified next to the expected ones, including the novel adapters Dok2 and ADAP, the tyrosine kinase Fer and the phosphatase SHP1 [75]. This study also revealed subtle differences between the CLEC-2 and GPVI signalling routes.

Another study of the protein composition of membrane lipid rafts found large similarities after platelet stimulation via CLEC-2 or GPVI [67]. Intriguing is an observed loss of cytoskeletal proteins in the rafts from the activated platelets. Extended analysis of the rhodocytin-stimulated phosphoproteome revealed a large panel of 0.4 k regulated phospho-tyrosine residues, among those multiple signalling and adaptor proteins, protein kinases and membrane-associated proteins [20]. Similar to the GPVI-induced activation, platelet stimulation via CLEC-2 relies on autocrine feed forward processes via released ADP and TXA_2_ [20,21]. Exactly which (phospho)proteome changes are dependent on these second mediators is still unclear.

## 7. Thrombin and Protease-Activated Receptors (PAR1, PAR4)

The serine protease thrombin cleaves coagulation factors, producing fibrin fibres from fibrinogen, and it also cleaves platelet protease-activated receptors (PARs) [76,77]. On human platelets, thrombin cleaves and activates the Gαq-protein-coupled receptors PAR1/4, while thrombin has an additional minor stimulatory role through its binding to the GPIb-V-IX complex [78]. PAR1 is the receptor operating at low thrombin concentrations, while PAR4 becomes activated at higher agonist concentrations [77]. The classical Gαq pathway leads to activation of phospholipase Cβ (PLCβ), which like PLCγ2 hydrolyses phosphatidylinositol 4,5-bisphosphate into the second messengers inositol trisphosphate (IP_3_) and diacylglycerol [5]. The formed IP_3_ induces via IP_3_ receptors the endoplasmic reticular release of Ca^2+^, while diacylglycerol stimulates protein kinase C (PKC) isoforms and the signalling mediator CalDAG-GEFI [79,80]. Granule secretion and aggregation are important responses of thrombin receptor-stimulated platelets. In addition, PARs can couple to Gα13 [81], which leads to activation of RhoA and Rho kinase (ROCK) via small GTP-binding proteins, and mediates platelet shape change and low-dose thrombin-induced aggregation [82].

After activation or ageing, platelets lose part of their constituents to the environment in several ways, i.e., by granular secretion, extracellular vesicle (microparticle) formation and receptor shedding [77]. Together, the lost proteins are termed as the releasate proteome [83]. Mass-spectrometric studies have examined the (granular) content of platelet releasates (Table S1). For a set of 0.1 k proteins, differences were reported between the PAR1- and GPVI-induced platelet releasates [84]. Among a broader set of 0.7–0.9 k proteins, ~10% seemed to be over- or under-represented in the platelet releasate from pregnant women [85,86]. However, the studies report various limitations, including low sample numbers and unclear clinical relevance (Table 1).

Next to the released secretome, the composition of PAR-induced extracellular vesicles (microparticles) was also analysed by mass spectrometry. For a set of 3.4 k proteins (0.4 k membrane proteins), it was found that thrombin induces the release of extracellular vesicles that are enriched in proteins sensitive to platelet activation, with an underrepresentation of granular proteins [87]. Examination of the platelet-derived extracellular vesicles from obese individuals showed expression changes in mitophagy and antioxidant defence proteins, when compared to non-obese subjects [88].

A pioneering paper in 2004 identified 62 differentially regulated protein features in PAR1-stimulated platelets [89]. A later report on a 3.4 k platelet proteome mentioned abundance changes in >20% of the proteins after PAR stimulation [18], a finding that will need further confirmation and explanation. In a focussed study, thrombin-induced Dab2 phosphorylation could be linked to platelet aggregation and ADP release [90]. Another report showed that inter-individual changes in the activated platelet proteome are related to miRNA levels [91]. This is one of the rare proteomic papers so far mentioning inter-individual variation.

## 8. Aspirin and Thromboxane A_2_

Several primary platelet agonists evoke release of the autocrine agent thromboxane A_2_, which is formed from arachidonate in phospholipids and is converted by the cyclooxygenase 1 (COX1)–thromboxane synthase complex [2,92]. Aspirin, the most common antithrombotic drug, irreversibly blocks the cyclooxygenase and hence the thromboxane synthesis. The platelet thromboxane receptor couples to the Gαq and Gα13 proteins, which results in PLCβ and ROCK activation, respectively [93]. The latter mediates the RhoA-dependent platelet shape change.

Interesting proteomic observations have been made in this context (Table S1). Using a label-free proteomic approach, platelets obtained from cord blood, which relatively poorly respond to thromboxane stimulation, express normal receptor levels, but are enriched in mitochondrial energy and metabolism proteins, including NDUFS1, NDUFA10, NDUFAS and NDUFY2 [94]. A perhaps accidental finding due to low sample size was that platelets from good and poor responders to aspirin treatment were differentiated in the level of carbonic anhydrase II [95].

Some reports have more extensively examined the effects of aspirin on platelets, considering that this drug can N-acetylate not only the thromboxane synthase complex, but also other many proteins. In an iTRAQ labelling study of the platelet glycoproteome, a small subset of the 0.8 k identified N-linked glycosylation sites was affected by aspirin treatment, among which was the secretory protein TIMP1 (metallopeptidase inhibitor 1) [63]. Furthermore, in a listing of 3.3 k acetylated residues, 6% showed aspirin regulation, with a higher acetylation state in the platelets from diabetic patients [96]. A pilot report stipulated that the lower aspirin effects on platelets from diabetics was linked to the glucose-suppressed glycation of in particular COX1 [97].

## 9. ADP Receptors and Platelet Inhibitors

ADP is another important secondary mediator that is released from granules after initial platelet activation, and ensures the formation of platelet aggregates and thrombi [5,98]. ADP itself activates platelets through the G-protein coupled P2Y_1_ and P2Y_12_ receptors to trigger different downstream pathways. The P2Y_1_ receptors support the first reversible phase of ADP-induced platelet aggregation, while the P2Y_12_ counterparts function to consolidate this aggregation. The only lowly expressed P2Y_1_ receptors evoke a Gαq-mediated signalling route via PLCβ, cytosolic Ca^2+^ elevation and PKC, such as that described for PAR1, but weaker in strength [80]. The P2Y_12_ receptors are Gαi coupled, activate PI3K forms and prevent adenylate cyclase to synthesize cAMP [98].

An ‘opposite’ pathway leading to platelet inhibition is triggered by the endothelial-derived prostacyclin (prostaglandin I_2_). Via a receptor on platelets, prostacyclin (or analogue iloprost) couples to Gαs (GNAS gene), which activates adenylate cyclase and increases cAMP [29,93]. The second messenger cAMP induces phosphorylation events by the broad-spectrum protein kinase A (PKA). In this way, prostacyclin can antagonise almost all platelet activation responses, including secretion, aggregation and procoagulant activity [2,99,100].

**Figure 3:**
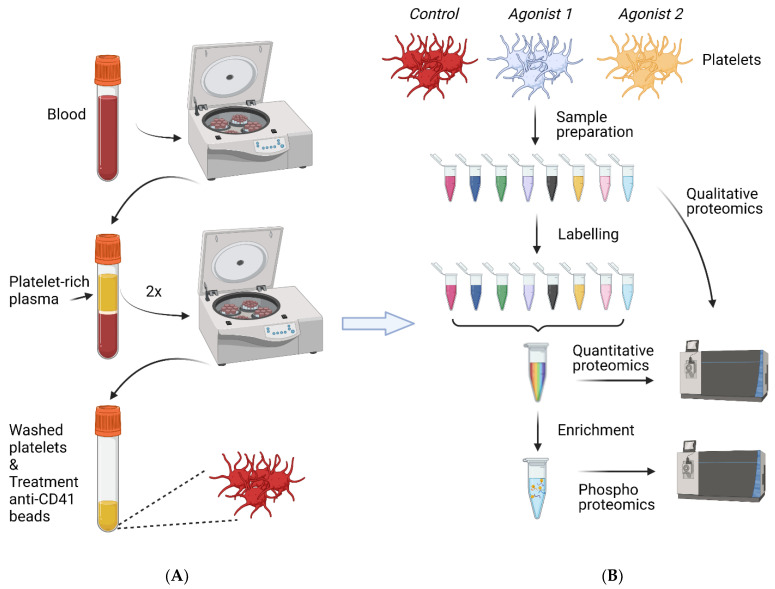
Global protocol of combined proteome analysis of (un)stimulated platelets. (**A**), Human platelet isolation from freshly drawn blood. Platelet-rich plasma (PRP) is collected by centrifugation and then re-centrifuged twice to obtain double-washed platelets. Removal of remaining leukocytes’ antibody-coated beads is advised. Platelet purity is determined by flow cytometry or microscopy. (**B**), Isolated platelets are stimulated with agonists as required. After lysis, proteins are fragmented by trypsin under well-controlled conditions, in this case in the presence of unique stable isotope labels. Pooled, labelled samples are fractionated, and peptides are resolved by LC-MS/MS analysis. For quantitative proteome information, reference peptides can be added. In case of phosphoproteome analysis or other post-translational modifications, an enrichment step is included for improved detection. An example protocol is provided in Box 1. Created with BioRender.com.

Proteomic-based studies were performed to better understand the antagonistic roles of ADP and prostacyclin (iloprost) by the analysis of (partly reversible) protein phosphorylations (Table S1). Using a quantitative labelling method for analysis of time-resolved phosphorylation changes (13% of 2.7 k phosphopeptides), it was concluded that platelet inhibition with iloprost is a multipronged process involving a broad spectrum of protein kinases and phosphatases, ubiquitinated proteins and many structural proteins [101]. Over 100 direct or indirect PKA targets were identified, revealing that platelets are inhibited by network-connected multiple signalling pathways [101]. Using the method of stable-isotope iTRAQ labelling (Figure 3), it was furthermore found that iloprost reverted 17% of the 0.4 k regulated phosphorylation sites (out of 3.6 k phosphopeptides) of ADP-stimulated platelets [102]. Dual-regulated phosphoproteins included signalling proteins and degranulation-regulating proteins, which in part were identified as targets of PKA or PKC.

Box 1 **Example workflow for bottom-up proteomic analysis of platelets.**

(i) Platelet preparation. Citrate-anticoagulated whole blood is centrifuged at 280× *g* for 10 min, to obtain platelet-rich plasma (PRP), the upper part of which is then centrifuged at 80× *g* for 10 min in the presence of anticoagulant medium [29] to pellet leukocytes. After pelleting the platelets (380× *g* for 10 min), the pellet is resuspended in buffer medium plus anticoagulant and apyrase. Another centrifugation step then gives washed platelets, which are resuspended in albumin-free buffer medium (≥5 × 10^8^/mL, ≥1.0 mg protein/mL). Leukocytes are counted preferably by microscopy, and immune depletion is applied if needed. The platelet suspension is left standing for 15–30 min. Samples (50–200+ µg protein) are stimulated by agonists as required, and stopped by addition of 4× concentrated lysis buffer (4% SDS, 150 mM NaCl, 50 mM Tris, pH 7.4, PhosStop added). Samples are frozen in liquid nitrogen and stored at −80 °C.

(ii) Peptide sample preparation. For in-solution digestion, the platelet proteins are reduced and alkylated in lysis buffer. Then, the lysis buffer is replaced by digestion buffer, and trypsin added to digest overnight. For relative quantification, isotopic reagents iTRAQ or TMT are used to label peptides. If needed, suitable enrichment methods are used, e.g., to concentrate phosphopeptides. RP-HPLC is commonly used to separate whole peptide mixtures, and hydrophilic interaction chromatography (HILIC) to separate phosphopeptides. Fractions are desalted to remove contaminants and detergents.

Recently, such studies were extended to nitric oxide, another major endothelial-derived potent platelet inhibitor [103,104]. Nitric oxide, as an unstable, membrane-permeable gas, increases platelet cGMP via guanylate cyclase, which produces protein kinase G (PKG) [104,105]. The formation of nitric oxide by platelets themselves via nitric oxide synthase is not likely [101]. Via a battery of cAMP/cGMP-dependent phosphodiesterases, the two cyclic nucleotides—and hence PKA and PKG activities—are ‘communicating’. The antagonism of platelet inhibitors (prostacyclin, nitric oxide) and platelet stimulators (ADP, thrombin, collagen) has led to phosphoproteomic analysis to identify protein phosphorylation events that determine the switch between inhibition and activation [106]. Herein, cross-talk signalling mechanisms were discovered, such as a feedback regulation of Syk by PKC [107]. It was also found that CLEC-2 activation triggers via ADP- and thromboxane the phosphorylation of a core set of signalling proteins (e.g., Src, PLCγ2) [20].

## 10. Platelet Proteomics of Patients with Platelet Defects or Cardiovascular Disease

Only limited proteomic studies have appeared on the changes in protein (modification) patterns of patients with platelet-related disorders (Table S1). The few examples are patients with Glanzmann’s thrombasthenia (lacking integrin α_IIb_β_3_, mutated *ITGA2B*), Scott syndrome (mutated phospholipid scramblase ANO6), X-linked thrombocytopenia (*GATA1* mutation), pseudohypoparathyroidism type Ia (PHP Ia, mutated GNAS locus) and Gray platelet syndrome (mutated *NBEAL2*, α-granule deficiency).

Targeted mass spectrometry confirmed that, for platelets from patients with type I Glanzmann thrombasthenia (a severe bleeding disorder), the level of integrin α_IIb_ was greatly reduced to <5% of control subjects [108]. In addition, plasma proteins endocytosed by integrin α_IIb_β_3_, such as fibrinogen, factor XIII, plasminogen and carboxypeptidase 2B, appeared to be downregulated when compared to the control platelets. Upregulated was the immunoglobulin receptor FcγRIIA, and in one patient the tetraspanin CD63 after FcγRIIA crosslinking, while the granular proteins were normal. Extensive global, phospho- and N-terminal proteome analysis was performed on platelets from a Scott patient (a rare, mild bleeding disorder), characterised by failure of agonist-induced phosphatidylserine exposure and procoagulant activity [109]. In the patient’s platelets, quantitative proteomics revealed a spectrum of 134 (6%) up- or downregulated proteins [28], including complete absence of the phospholipid scramblase anoctamin-6 and low calpain-1 protease (regulating platelet morphology changes) (Table 2). Interestingly, the cell-volume-regulating protein aquaporin-1 was upregulated, putatively as a compensatory mechanism.

A mutation in exon 4 of the *GATA1* gene is associated with X-linked thrombocytopenia with thalassemia (OMIM 314050), which is a severe bleeding disorder. Quantitative proteomics of the platelets from five male patients revealed 83 altered proteins [30]. Among these were COX1 plus several cytoskeleton and proteasome proteins (Table 2). In comparison, in platelets with mutant GATA binding factor 1, more than 300 proteins were proposed to be differentially expressed in comparison to control subjects [110].

In 5 out of 47 Gray platelet syndrome patients (a milder bleeding disorder) with new variants in *NBEAL2*, 123 platelet proteins were mostly downregulated, with the majority being α-granule-associated and cargo proteins at unaltered mRNA expression levels (Table 2) [31]. Markedly, plasma proteins related to immune responses and inflammation were upregulated, which suggested the presence of an immune defect in these patients as well. In the platelets from five patients with PHPIa (Albright hereditary osteodystrophy, a disease of hormonal resistance and abnormal postures, no bleeding), quantitative phosphoproteomics revealed 0.5 k iloprost-regulated phosphorylation sites [29]. In agreement with a loss-of-function of Gαs in the patient’s platelets, a panel of 51 phosphorylated proteins was identified that showed a consensus PKA phosphorylation site and that was altered in most of the patients (Table 2).

In addition to these rare congenital abnormalities, few proteomic studies have examined platelets from patients with somatic mutations (cancer) or genetically less well-defined diseases. In a study on platelets from 12 patients with early-stage cancers (in comparison to healthy subjects), quantitative proteomic analysis indicated disease regulation of a wide variety of 85 (3%) proteins (Table 2), the majority of which normalised after surgical resection [19]. Some of the regulated proteins may be useful as biomarkers for such cancers. This area requires further attention.

Analysis of the platelet proteome of patients with progressive multiple sclerosis showed elevated levels of plasma proteins such as fibrinogen and α_2_-macroglobulin [111], pointing to increased endocytosis or stickiness of the patient platelets. In patients infected with dengue virus, platelet quantitative proteomics identified about 0.3 k regulated proteins [112]. With the aim to find a biomarker related to Alzheimer’s disease, proteomic analysis of platelets from patients with mild and severe cognitive function revealed 360 differentially regulated proteins. Four of these (PHB, UQCRH, GP1BA and FINC) were able to distinguish patients from healthy controls [113]. However, the link to disease is still unclear.

A few studies examined the platelets from patients with cardiovascular diseases (Table S1). Differentially regulated proteins were identified in the platelets from two groups of patients with (acute) coronary syndrome; there were, in particular, signalling, glycolysis and cytoskeletal proteins [114,115]. In patients experiencing ST-elevation myocardial infarction (STEMI), gel-based proteomics identified 16 altered proteins in platelets that were collected at the intracoronary culprit site, including integrin α_IIb_ and thrombospondin-1, in comparison to the circulating platelets [116]. Additionally, in STEMI patients, platelet phosphoproteomic analysis revealed an increase in key tyrosine phosphorylations in response to GPVI stimulation, which raised the possibility of GPVI as an antithrombotic target in STEMI [84,116]. Only a few altered proteins were found in the platelet releasate of patients with stable angina pectoris [117], and in whole platelets from patients with lupus anticoagulant-related thrombosis [118], along with evidence for increased platelet activation. Additionally, in hyperlipidaemia, the platelet proteome was found to be markedly unchanged [119]. Semi-quantitative workflows were developed to find platelet-related protein biomarkers in cardiovascular disease [120].

## 11. Practical and Technical Considerations

Platelet preparation. In current platelet proteomic research, washed platelets are commonly used. As indicated in Figure 3, the platelet isolation protocol is usually based on a series of centrifugation steps to separate platelets from other blood cells and plasma. In Box 1, we provide a validated protocol based on our own experience. However, given the presence of an extended open canicular system in platelets in direct contact with the blood plasma, residual plasma proteins are invariably seen in most listed platelet proteomes [11]. The reviewed papers show a wide variety of platelet purities, platelet concentrations and amounts of proteins used per analysed sample (Table S1). Furthermore, the procedure to use platelets at a resting state is important; this can be evaluated by flow cytometry (e.g., checking for integrin α_IIb_β_3_ activation and P-selectin expression) or by parallel-reaction monitoring (PRM) tests [102]. It is of no doubt that all these variables can influence the composition and size of an established platelet proteome, and that standardisation is therefore needed (Box 1). The agonists and ways to activate platelets are other matters of variation, as seen between papers, in that most agonists indirectly trigger secondary pathways via release of ADP, TXA_2_ and other autocoids [2]. This is very likely to affect (time courses of) phosphorylation outcomes [102].

The complexity of platelet proteome analysis is increased by a wide variety of post-transcriptional protein modifications (protease processing, acetylation, acylation, phosphorylation and glycosylation) [8,9]. In our experience, new mass spectrometry acquisition techniques such as data-independent acquisition make it easier to quantify low-abundance (post-translated) protein forms.

Peptide sample preparation. At present, sample preparations for bottom-up proteomics analyses (no prior gel separation) is the most common technique. For the collection of trypsin-digested peptides, filter-aided sample preparation, S-trap-based digestion methods and ethanol precipitation are preferred [121,122]. Novel proteomic approaches at the horizon are a nanodroplet processing platform for analysis of small cell numbers [123].

For higher throughput purposes, label-free analysis can be performed [13], where unlimited sample numbers can be compared at the proteome level. Another ratioed method makes use of stable isotope sets, such as in iTRAQ- and TMT-based quantification, where up to 16 (phosphopeptide) samples are pooled together [29,63]. Prior enrichment methods for phosphopeptides relied on metal oxide affinity chromatography with titanium dioxide (TiO_2_) beads [24,28,102]. An alternative is provided by immobilized metal affinity chromatography (IMAC) with liquid handling systems, which allow phosphopeptide enrichment of up to 96 samples at the same time [124].

Data analysis. The most commonly used strategy for proteomic data analysis is searching against established fragmentation spectra databases. A helpful overview of current bioinformatics methods for protein identification and quantification is given in [125]. An important step in the analysis is data normalization, where corrections according to specific rules are applied to remove inconsistent data points, followed by statistical tests (checking for false discovery rates). Regarding phosphoproteomics, data normalization is also necessary because the apparent regulation of a phosphorylation site should not be caused by an altered protein abundance [126]. Special algorithms have to be employed for assessing the phosphorylation position and the responsible kinase of a phosphorylated peptide [29].

Current limitations. Many of the publications indicated as a limitation the use and comparison of only a few platelet samples (Table 1). This has made it difficult to draw conclusions on inter-subject variation, even when comparing healthy subjects with patients. Technical limitations include low peptide coverage linked to low protein abundance, complex spectral data analysis, missing (hydrophobic) peptide sequences and unclear function of many discovered proteins [8].

## 12. Future Perspectives and Challenges Ahead

Over the last 20 years, the field of platelet proteomics has evolved rapidly, from the early 2D gel electrophoresis studies to the more recent bottom-up-based ones. The evolution of recent generations of mass spectrometers has allowed protein quantitative studies and the elucidation of complex phosphorylation patterns of proteins with often still unknown functions. Enrichment steps, for instance, to concentrate negatively charged peptides from trypsin-treated lysates, and advanced sample preparation protocols are now bypassing the earlier gel separation steps.

As shown in this review, the new proteomic methods have led to a gradual enlargement of the human molecular platelet (phospho)proteome, thus identifying so far up to 6 k identified proteins (3.7 k with copy numbers), which however are only partly linked to known platelet functions. The platelet phosphoproteome contains up to 5 k phosphorylation sites with many sites altered upon platelet stimulation (via GPVI, PARs, CLEC-2, ADP) or platelet inhibition (with iloprost). The regulated Ser/Thr and Tyr phosphorylation sites included not only the known or expected signalling proteins, but also a multitude of poorly understood signalling, regulatory, structural and metabolic proteins. Diverse sets of previously known and new proteins were found to be altered in pathological situations, but the ‘regulation’ of these was mostly not confirmed in independent studies. As described above, advances in the proteomic field have also led to the identification of (novel) platelet proteins as potential biomarkers for disease.

Accordingly, proteomics has been developed as a fundamental tool for platelet research. On the other hand, it is time now to consider how to turn this field from the phase of discovery into the phase of biological and (pre)clinical application. The analysis of 70 papers in the present review has taught us that for this transition, a number of challenges are ahead. These can be grouped into the following eight points.

Despite the broad heterogeneity in mass spectrometry and spectral analysis methods, the precise composition of the ‘normal’ human platelet proteome is still unclear. While the theoretical proteome recently could be deduced from the genome-wide platelet transcriptome of 14.8 k protein-encoding transcripts [11], published papers so far show only parts (up to 5 k) of these proteins, with often limited overlap between the various lists. A coordinated multi-laboratory effort will be needed to demarcate the achievable human platelet proteome. In this respect, an update will also be needed of the 2014 reported list of protein copy numbers per platelet [24].

The outcome of quantitative platelet proteomics greatly depends on the method and purity of sample preparation, including the platelet concentration, activation state and all sample processing. Even between recent papers, the described procedures highly deviate. The same holds for platelet ‘products’ such as the releasate, secretome and extracellular vesicles. We envision that inter-laboratory standardization is needed for better comparison of new study results. As a start, we provide an experience-based protocol (Box 1).

With the platelet proteomic field moving from protein discovery to assessment of protein composition, there is an urgent need for reliable and consistent quantification methods for (core sets of) platelet proteins. In only a few published studies, the judgements on up- or downregulation of proteins were validated by independent means. Hence, in papers comparing subjects or patients, information is missing on the consistency and reproducibility of the found differences. As a solution, we propose duplicate or triplicate workflows on parallel platelet samples, and a consistent use of internal standards.

Analysis of the platelet phosphoproteome has revealed an unexpected wealth of time-dependent regulated protein phosphorylation sites. However, the precision and meaning of the changes in many proteins are unclear, even in stable isotope (iTRAQ, TMT) comparisons. On the other hand, the phosphorylation analysis of platelets from obese subjects and several genotyped patients does provide novel insight into the dysregulation of platelet functions in these disorders.

Several studies report on altered proteomes of stored platelets for transfusion and of released platelet products. The work mostly aims to provide new biomarkers for platelet product quality or the platelet activation state (in vivo). For many proteins with an assumed biomarker role, the physiological functions in platelets are unclear, which asks for a more sophisticated protein function analysis than the conventional pathway analyses (e.g., by Gene Ontology). A recent classification system of all proteins present or expected in platelets may help to achieve this goal [11]. Once a biomarker is confirmed, at a later stage mass spectrometry can be replaced by cheaper, immune-based or flow-cytometry methods in larger (clinical) studies.

The published studies with patients so far are all limited by low sample sizes, so that common inter-subject variables such as blood cell traits, gender, age and health history are not examined. New high-throughput analysis, using label-free quantification methods in combination with data-independent acquisition, will allow the comparison of multiple platelet samples at the same time, which is conditional for these clinically related questions.

Application of platelet proteomics in the diagnostic laboratory is a challenge due to the expensive and complex requirements in terms of sample preparation and equipment. The additive value of clinical proteomics likely is highest for patients with complex disorders where genetic analysis fails, e.g., in cases of not-understood bleeding, or in metabolic or other systemic diseases. Additionally, otherwise, to understand the action mechanisms of new (antithrombotic) drugs.

In general, we propose that, for the field to move forward, common guidelines should be established that help to improve the inter-lab reproducibility of platelet preparation, proteomic sample processing and complex data analysis. This has also been acknowledged at the 2021 ISTH Congress.

## Figures and Tables

**Table 1:** Proceedings and characteristics per section of published proteomic studies regarding platelet purity, sample size, types of proteomes analysed and reported study limitations. For details per study, see Table S1. Abbreviation: PRM, parallel-reaction monitoring.

Section	Year	Purity Checked	Sample Size	Type of (Sub)Proteomes	Reported Limitations	Pathway (GO)
3-Basic	≥2011	6/12	20–250 µg	platelets, granules, palmitoylation, methylation	unclear relation to platelet functions, low sample number	8/12
4-Ageing	≥2012	3/12	4–500 µg	stored platelets, N-terminome, extracellular vesicles	unclear relation to platelet functions, low sample number	7/12
5-GPVI	≥2010	8/11	150–2500+ µg	platelets (label-free), phosphorylation (TiO_2_), acetylation, PRM targeted, ubiquitylation, releasate	limited protein recovery, sample pooling	7/11
6-CLEC-2	≥2012	2/2	150 µg	platelets, phosphorylation	limited protein recovery, second mediator interference	2/2
7-PARs	≥2015	5/13	24–150 µg	platelets (label-free), phosphorylation (TiO_2_), releasate, extracellular vesicles	sample pooling, low sample number, leukocyte contamination, clinical relevance unclear	12/13
8-ASA	≥2017	2/5	5–100 µg	platelets, acetylation, glycosylation	unclear clinical relevance	3/5
9-ADP/INH	≥2014	3/3	100–800 µg	platelets, phosphorylation (TiO_2_)	low sample number, unclear function of phosphorylation	2/3
10-PAT	≥2010	7/15	40–600+ µg	platelets (label-free), targeted, phosphorylation, N-terminome	low patient number, inter-patient variation, unclear relation to platelet functions	8/15

**Table 2:** Selection of protein changes in published platelet proteomes. Confined to papers showing abundant regulated proteins in platelet (fractions from) patients. Italic: plasma or secretory proteins [11].

Aim of Study	No. of Regulated Proteins	Selection of Regulated Proteins	Ref.
3-BASIC. Proteomes of large and small platelets (1 subject)	80 up or down (9%)	ADP-ribosylation factor 1/3, GTP-binding protein SAR1a, guanylate cyclase soluble subunit α3, voltage-dependent anion channel protein 3, serotransferrin, immunoglobulins, haptoglobin, hemopexin, α1-antitrypsin, vitronectin	[27]
5-GPVI. Platelet proteome in Scott syndrome (1 patient)	134 up or down (6%)	anoctamin 6, annexin A5, calpain 1, protein S100-A8/9, channel aquaporin-1, pregnancy zone protein, myeloperoxidase, serine-pyruvate aminotransferase, platelet glycoprotein 4, cAMP-dependent protein kinase IIβ regulatory subunit	[28]
10-PAT. Platelet iloprost phosphoproteome of PHP Ia patients (6 patients)	51 up or down (11%)	phosphorylation of protein kinase A consensus sites: inositol triphosphate receptor associated 1/2, bridging integrator 2, vasodilator-stimulated phosphoprotein, coiled-coil domain-containing protein 9, claudin 5, consortin, Grb2-associated binding protein 2.	[29]
10-PAT. Platelet proteome in X-linked thrombocytopenia (5 patients)	83 up or down (4%)	prostaglandin G/H synthase 1, solute carrier family 35 member D3, carbonic anhydrase 2, peroxiredoxin 1, tubulin-tyrosine ligase-like protein 12, spectrin α-chain, nexilin, E2 ubiquitin-conjugating enzyme, proteasome subunit α/βtype 4, heat shock 70 kDa protein 1b	[30]
10-PAT. Platelet proteome in Gray platelet syndrome (5 patients)	123 up or down (% n.d.)	neurobeachin-like protein 2, SPARC, serglycin, latent-transforming growth factor β-binding protein 1, platelet basic protein, thrombospondin-1, platelet factor 4, multimerin-1, von Willebrand factor	[31]
10-PAT. Platelet proteome of patients with early-stage cancer (12 patients)	85 up or down (3%)	mitochondrial deoxyribonucleotidase and 39 S ribosomal protein, V-type proton ATPase subunit S1, myomegalin, serine/arginine repetitive matrix protein 2, CD34, peptidoglycan recognition protein 1, nuclear ribonucleoprotein A1, histone H2B type 1J, neurochondrin, calmodulin-dependent protein kinase type 1D	[19]

## Supplementary Materials

The following are available online at https://www.mdpi.com/article/10.3390/ijms22189860/s1.
